# Toward Effective Technology Support in DCTs: Insights from the Trials@Home Proof-of-Concept Trial RADIAL

**DOI:** 10.1007/s10916-026-02352-x

**Published:** 2026-03-05

**Authors:** Theresa Weitlaner, Dimitrios Giannikopoulos, Bart Lagerwaard, Hannes Hilberger, Bernhard Neumayer, Mira G. P. Zuidgeest, Sten Hanke

**Affiliations:** 1 Institute of eHealth, FH JOANNEUM University of Applied Sciences, Graz, Austria; 2 HEALTH - Institute for Biomedical Research and Technologies, JOANNEUM RESEARCH Forschungsgesellschaft mbH, Graz, Austria; 3 Bayer AG, Wuppertal, Germany; 4 Julius Center for Health Sciences and Primary Care, University Medical Center Utrecht, Utrecht, The Netherlands; 5 Gottfried Schatz Research Center, Division of Medical Physics and Biophysics, Medical University of Graz, Graz, Austria; 6 BBMRI-ERIC, Graz, Austria

**Keywords:** Decentralized clinical trials, Key performance indicator, Knowledge base, Helpdesk, Technology support system, Ticketing system

## Abstract

Decentralized clinical trials (DCTs) increasingly rely on digital tools such as wearable devices, mobile applications, and online platforms to enable remote data collection and participant engagement. While these technologies offer opportunities for greater convenience and continuous data capture, they also introduce operational complexity and require comprehensive technical support for participants, clinical site staff, and study teams. This paper reports on the design, implementation, use, and evaluation of a dedicated support framework in the European proof-of-concept trial RADIAL, part of the Trials@Home project, aimed at identifying effective components and operational practices for supporting DCTs. The framework included an open-source ticketing system for structured issue management, a restricted-access knowledge base (KB) with training materials and troubleshooting guides, and governance processes for escalation and quality monitoring. Key performance indicators were derived from system databases to track ticket volume, resolution times, and KB usage. Over the trial’s 17-month reporting period, 169 tickets were submitted across 90% of active clinical trial sites, with device-related problems accounting for 46% of requests and study app queries for 17%. Half of all tickets were resolved within three days, and most required two to six replies. The KB logged 696 searches and 4,350 article views, with highest engagement around training materials and device-related instructions. Findings indicate that a successful support framework for DCTs requires a combination of ticketing, accessible documentation, live support options, and continuous governance. Lessons from RADIAL underscore the importance of multilingual support, proactive training, and flexible workflows to effectively mitigate operational complexity and ensure reliable trial conduct.

This trial was registered with identifier NCT05780151 in clinicaltrials.gov and under 2022-500,449-26-00 in the Clinical Trials Information System (CTIS) clinical trial database.

## Introduction

Decentralized clinical trials (DCTs) often rely on a complex integration of diverse technological components in their effort to move trial activities to a participant’s own and surrounding environment. These trial setups are typically modular, combining hardware and software solutions from various vendors to create customized systems that support remote study conduct. In addition to conventional trial technologies, DCTs increasingly incorporate tools such as wearable sensors, remote monitoring devices, study smartphone applications, and online platforms [5, 16, 19]. These innovations, while enhancing data collection [13] and potentially participant adherence [8, 12] also introduce new challenges. They often require higher levels of technological literacy and readiness from participants, clinical research associates (CRAs), and clinical site personnel, who must be equipped to handle troubleshooting and system navigation [2]. As a result, sponsor-provided technical support for sites, CRAs, and participants is essential and differs significantly from that in conventional clinical trials. It must cover a broad range of needs, including hardware functionality, software compatibility, user access management, and system maintenance: Frequent challenges related to hardware, software, and user access have been reported by Valdez et al. [18], whereas Perez et al. [11] highlight challenges around authentication and platform compatibility. In addition, maintaining independence from specific browsers or operating systems necessitates a continuous need for system updates and adaptation [11]. Effective support in this context relies heavily on clearly defined communication protocols – such as real-time chat, ticketing systems, and collaborative document sharing – and well-maintained, accessible documentation [7, 15]. In other studies, telehealth implementations have demonstrated the importance of training, iterative system development, and active clinician involvement to overcome technology-related challenges [1, 17]. Valdez et al. [18] describe common technical issues managed through a service desk model, addressing hardware, software, and access concerns. Several studies emphasize the importance of timely access to study-related data for investigators, clinicians, and data managers, alongside streamlined document workflows [14, 15]. Timely and accurate responses to technical queries are critical, as delays can disrupt study procedures [6, 11]. Training and preparation for site staff are equally important [1, 17], and dedicated resources should be allocated to manage the increased support demand [6]. Furthermore, ongoing quality monitoring and iterative development based on user feedback are vital for refining systems over time [11, 15]. Across multiple studies [11, 14, 15, 17], usability and intuitive interface design are consistently identified as key factors influencing technology adoption and user satisfaction.

Various models of support have been implemented in DCTs, ranging from 1) real-time human support [7, 18], to 2) asynchronous systems such as ticketing services and help desks [11, 15]. The latter document the use of electronic portals that streamline sponsor–investigator communication but can also raise usability concerns.

Support approaches are categorized into real-time human interaction, asynchronous human support, electronic communication platforms, structured technology training, and data management tools. These services may be organized through centralized models, such as unified ticketing systems and electronic portals, or distributed frameworks involving service desks with real-time or on-demand support. Integration with clinical workflows is a recurring theme and emphasizes the need for support systems to align with the routine practices of both participants and staff [14, 15, 17].

To accompany the RADIAL trial [19], conducted as part of the Trials@Home project, the aim was to design, implement, and test a technology support system addressing the key DCT-related challenges described above and to evaluate it in a real-world clinical setting. The RADIAL support system included an asynchronous ticketing system for issue tracking, scheduled on-demand human support, and an online wiki-style knowledge base (KB) that provided stakeholder-specific training materials. To better understand RADIAL’s requirements for a helpdesk and the solution implemented, both Trials@Home and RADIAL are described in the following.

### The Trials@Home Project

The objective of the Trials@Home initiative is to identify opportunities for conducting clinical trials closer to the participant’s everyday environment. The project is driven by the goal of transforming the design and conduct of clinical trials. The aim is to develop and pilot standards, tools, and recommendations to guide the future definition of DCTs in practice. Innovative approaches should minimize participant burden, eliminate geographical constraints, and improve study data reliability.

A patient-centric design enables participants to contribute to research from their home, thereby alleviating logistical and time-related stress associated with on-site visits. The integration of cutting-edge digital health technologies (DHTs), including mobile medical devices and a customized mobile study app, enables remote data collection. These devices allow continuous, real-world monitoring, generating datasets that more accurately reflect the intervention’s effectiveness in everyday settings while also promoting participant engagement and adherence. The relocation of most or all trial activities to participant’s homes supports greater inclusivity and equitable access to clinical trials.

Trials@Home employs a co-creative multi-stakeholder approach, with the overarching goal of establishing a network of clinical investigators, engineers, and participants, that fosters seamless collaboration. Ultimately, by blending existing and novel techniques for use in DCTs, the Trials@Home research aims to validate these concepts in a proof-of-concept trial, called RADIAL.

### The RADIAL Trial

RADIAL, an acronym for Remote And Decentralised Innovative Approaches to cLinical trials, is a European proof-of-concept trial that aims to assess the feasibility and acceptability of conducting future clinical trials from participants’ homes rather than at clinical (research) centers. RADIAL investigated the feasibility of DCT models by comparing fully remote and hybrid designs with a conventional approach. The evaluation focuses on scientific and operational dimensions, including participant recruitment, retention, diversity, satisfaction, and cost, alongside safety oversight, treatment adherence, and data quality. The secondary objective examines whether therapeutic efficacy of Insulin Glargine 300 U/mL, a long-acting insulin intended for individuals with Type 2 diabetes mellitus, is maintained across trial arms with varying degrees of decentralization. Consequently, the trial aims to respond to a methodological research question independent of commercial interests. [19]

Participants were recruited from multiple sites across the United Kingdom, Poland, Spain, Italy, Denmark, and Germany. The treatment period lasted 24 weeks. A bring-your-own-device (BYOD) methodology restricted to smartphones was employed, with all other devices supplied by the trial.

Using onsite recruitment methods, 100 participants were recruited into Part A, comprising the conventional and hybrid treatment arms. Participants in the conventional study format experienced primarily onsite trial interactions, while the hybrid arm involved a combination of home nurse visits and on-site visits. Exemplifying the full potential of DCTs, the fully decentralized treatment arm was carried out entirely remotely, with participants conducting all trial activities from their homes. Eight participants were recruited online and engaged solely with decentralized trial elements (remote arm, Part B). These include remote consenting, home nursing visits, remote monitoring of compliance with the study protocol through DHTs (smart injection cap, app for participant-reported outcomes and medical events), direct-to-participant shipping of medication and study materials, and self-conducted blood finger-prick at home. A complete overview of recruitment numbers and activities is available in a separate publication [10]. All elements of the support system (KB, ticketing system) were available to all clinical site personnel and CRAs at all participating trial sites, independent of their actual recruitment into the study arms.

### The Need for Robust Technology Support

In consideration of the RADIAL trial’s design, the integration of three arms with different levels of decentralization (conventional site-based, hybrid, remote) demonstrates the necessity for a technology support system capable of adapting to each arm [5].

The BYOD approach further increases the level of effort required to ensure that participants can install, access, and configure all study-related technologies on their own devices without losing valuable participant-reported data, thereby ensuring adherence to the study protocol.

**Fig. 1:**
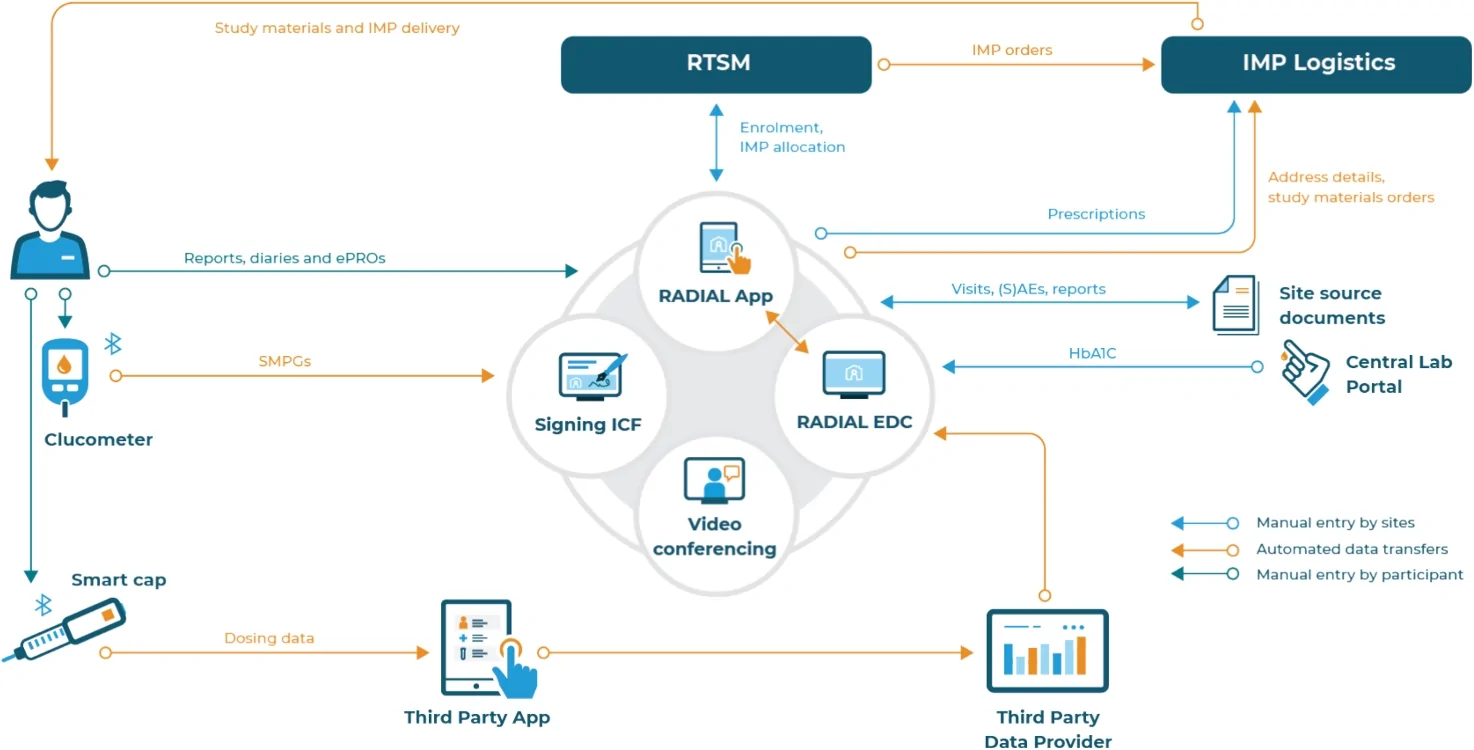
Overview of systems for the remote arm of the Trials@Home proof-of-concept trial RADIAL. RTSM, Randomization and Trial Supply Management System; IMP, Investigational Medicinal Product; EDC, Electronic Data Capture system; ICF Informed Consent Form; ePROs, electronic Patient-Reported Outcomes; (S)AEs, (Serious) Adverse Event Reports; SMPGs, Self-Measured Plasma Glucose; HbA1c, glycated hemoglobin

A thorough examination of the technology framework diagram (Fig. 1) reveals that the remote arm of the RADIAL trial alone involves multiple systems and data flows. In the context of RADIAL, as is often the case in other DCTs, technology solutions are provided by multiple hardware and software vendors, each with varying levels of integration. This fragmentation requires a comprehensive and well-coordinated technical support system that oversees the study in its entirety and ensures seamless operation across all components. The involvement of multiple stakeholders, in the RADIAL case, a consortium including the sponsor, study teams, participants, vendors, and site users from different countries adds further complexity, making a structured and well-governed technical support system even more critical to successful DCT operations.

A sophisticated technology support system is therefore pivotal for ensuring the safety, quality, and validity of DCTs, while minimizing participant and site burden. In addition, analyzing helpdesk requests, as well as solutions and processing time can help to determine and further enhance the acceptability of future DCTs.

### Objectives

This paper presents the setup and evaluation of the implemented support system for the RADIAL proof-of-concept trial, detailing its operational processes and performance outcomes. By analyzing data from the ticketing system, the most frequently encountered issue types during the trial were identified to estimate the effectiveness of the support model. Insights from the RADIAL experience, aligned with the literature and emerging technological trends, are synthesized into a set of functional and operational recommendations.

## Methods

The RADIAL support system was designed to assist all stakeholders involved in the trial, particularly in the context of a DCT setup with multiple technology systems in place. To achieve this, the system was set up to provide a structured ticketing process for issue tracking and resolution, a KB containing training materials, frequently asked questions (FAQs), and user manuals, all hosted and maintained throughout the RADIAL trial. In addition, the system was implemented using an open-source solution to minimize costs and enable study-specific customization.

### Technical Setup of the RADIAL Technology Support System

Given the wide availability of potential software packages, several different solutions were tested through local server installation, request simulations, feedback loops, and content creation to evaluate workflow suitability. Ultimately, the open-source platform UVdesk (https://www.uvdesk.com/) system was chosen for user support. It handles tickets via a central e-mail address, allows the definition of user groups, includes a search function for the KB, and provides an adequate overview of a ticket’s history. The user groups can be used to restrict access to certain content as well as to assign certain topics to specific teams. Tickets can be forwarded to specific members, even external persons if necessary.

Given the inclusion of sensitive trial and proprietary vendor materials, an essential requirement was the restriction of KB content from public access; therefore, the system – using the open-source nature of the solution – was extended by a custom login feature: Access to the KB frontend was set up using country-specific credentials, supplemented with additional accounts for the CRAs across all countries and the support team. The backend and ticketing system required individual login credentials with role-dependent permissions. Further details on the customization of the support system and role specifications are provided in [4]. UVdesk stores its content in databases, which allows to access the content via SQL requests. This was used to log search queries, export tickets to derive content for FAQs or to visualize time-to-solution.

### Operationalization and Governance Tools

The technology support system provided CRAs and site staff with quick access to essential information, empowering site staff to effectively assist trial participants. The ticketing system enabled users to report issues and request assistance. A dedicated support team of ticket agents provided direct support, with multilingual assistance available when needed. The system also coordinated with third-party vendors, ensuring timely resolution of issues related to specific technology components.

**Fig. 2:**
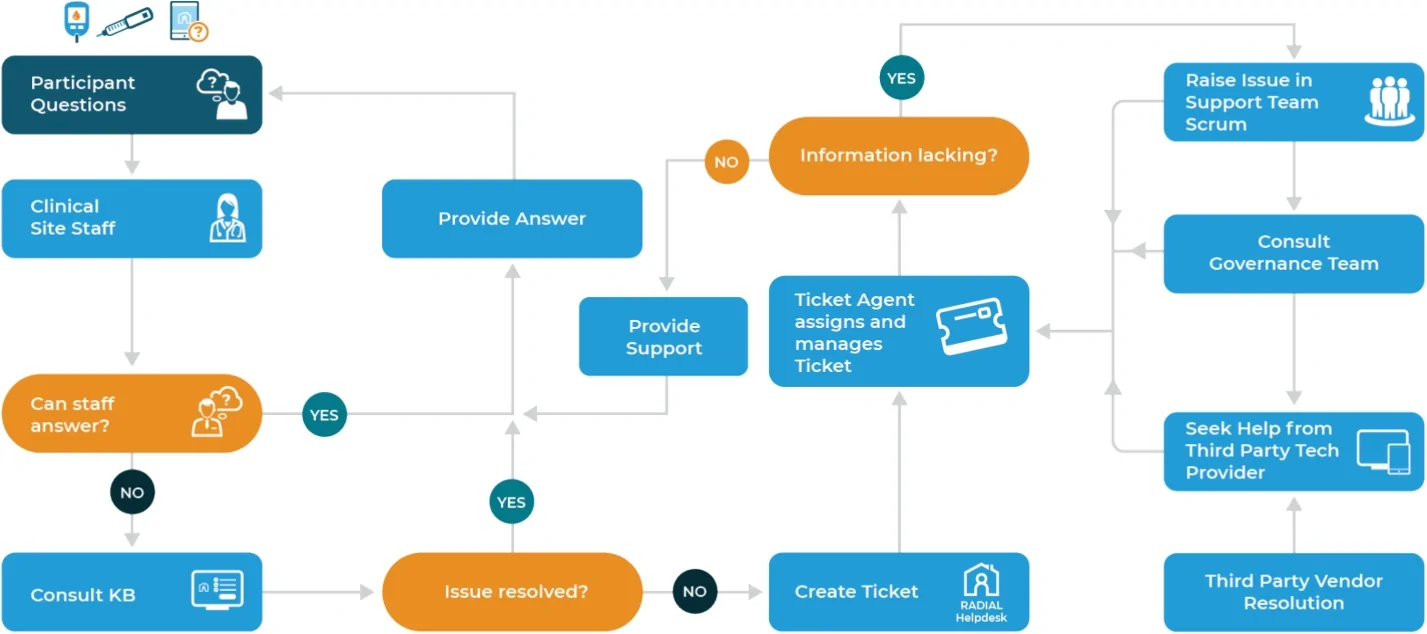
Intended workflow within the technology support framework in RADIAL; KB, Knowledge Base

In RADIAL, the intended workflow (Fig. 2) using the system was as follows: Participants directed questions to clinical site staff. If they could not provide an immediate answer, clinical site staff supported by CRAs consulted the KB either directly via topic folders or through free-text search. The KB contained manuals, training materials, videos, and guidelines, and unresolved issues were escalated by creating a ticket through the helpdesk. Ticket agents adhering to a coverage plan were working under additional support, forming the RADIAL support team; if information was lacking, they followed-up with the site or CRAs, raised issues in weekly scrum meetings, consulted the governance team, or sought help directly from the technology provider. The third-party vendor resolved such cases in their own system and informed the RADIAL helpdesk to ensure proper documentation and closure.

In the open-source version of the UVdesk, the ticket request form did not allow users to add additional recipients of ticket updates. If additional contacts were required, users could add recipients’ details to their message and the ticket agent in charge would manually add them as “collaborators” to the ticket. After this step, responses were automatically dispatched to all collaborators, and all responses were logged in the ticketing system. The initiation of each response triggered an automatic e-mail notification to the assigned ticket agents and collaborators ensuring seamless information flow. This interactive process continued until the ticket creator’s inquiries were fully resolved.

In the RADIAL ticketing system, requests were tracked through standardized status categories. Newly submitted tickets were marked “Open” by default until assigned, after which they became “Pending” while under review or investigation. Once a response or solution was provided, tickets were marked as “Answered”, with follow-up allowed before conversion to “Resolved” if no reply was received within 15 days. Tickets directly related to study issues were classified as “Resolved”, whereas unrelated issues were designated “Closed”.

Ticket categorization was initially set by the ticket creator, who selected from predefined labels – such as platform, app, device, or third-party technology – to aid triaging. Support agents or collaborators could update the category when needed. New types were added during the study; for example, “Schedule Live Support Session” enabled phone or video assistance for issues beyond chat or email. Ticket creators also assigned a priority level (low, medium, or high) to guide resolution according to urgency and impact.

The ticket agent support team convened weekly scrum meetings to analyze ticket trends, identify training gaps, and escalate unresolved or critical issues to the governance team in line with established procedures. All helpdesk tickets, regardless of status, were consolidated into a global issue tracker. The tracker recorded key attributes for each ticket, such as unique identifier, participant identification, site identification, current status, and assigned ticket type. It also documented the initial ticket submission message, a concise issue description derived from scrum meeting minutes, and, where applicable, the corresponding resolution details.

The governance team, responsible for overseeing the technological components of RADIAL, and including quality assurance specialists focusing on participant data integrity and risk management, jointly reviewed and updated the tracker during weekly governance meetings. These reviews addressed unresolved issues and identified potential systemic risks. As part of this process, the governance team reclassified tickets into predefined categories: “Bugs and Errors”, “Process-related”, “Technical Support”, “User Access and Permissions”, “General Inquiries and Information”, and “Not Relevant for RADIAL”.

### Data Sources and Analysis Methods

The open-source version of UVdesk does not include a built-in reporting or analytics module. Consequently, key performance indicators (KPIs) – including issue count, response frequency, and average resolution time – were obtained directly from the UVdesk database via SQL queries. These metrics were then aggregated into monthly and on demand reports to provide actionable insights into system performance and efficiency.

**Fig. 3:**
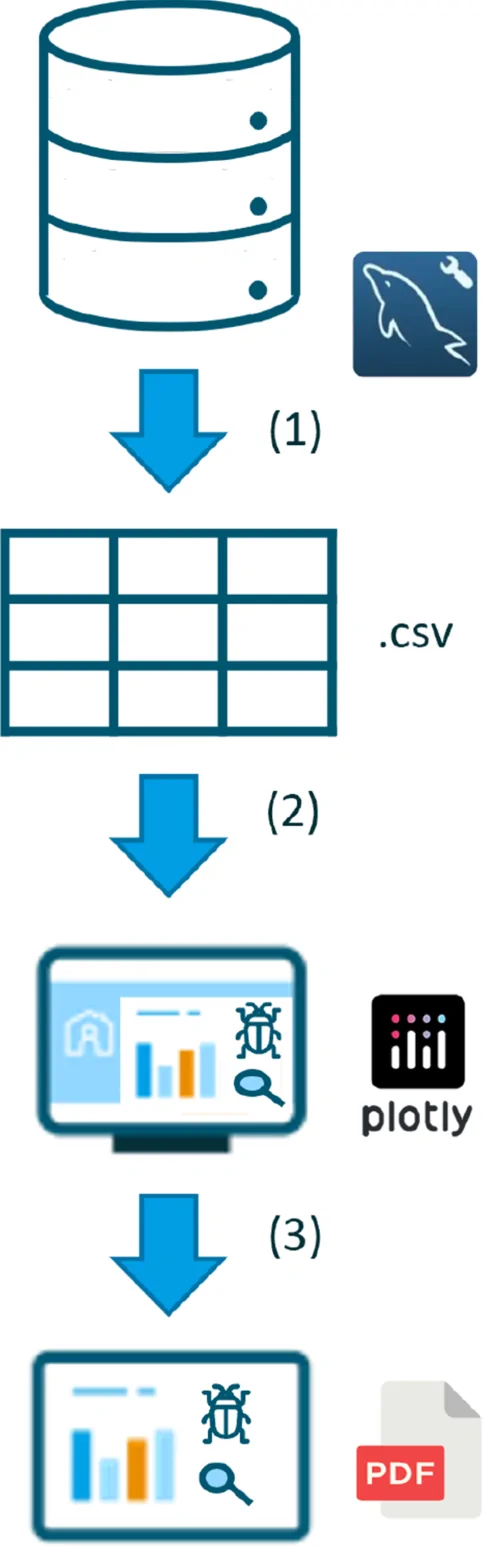
Workflow for key performance indicator generation: (1) Data export from the ticketing system’s relational database via SQL queries in MySQL Workbench to CSV format; (2) Preprocessing and visualization using Python and the Plotly Dash web application; and (3) Compilation into a stakeholder-oriented PDF report. Adapted from [4]

The generation of KPIs from the RADIAL ticketing system and the KB followed a three-stage process. First, relevant data were exported from the ticketing system’s relational database. Second, the extracted data underwent preprocessing – such as calculating average resolution times – using Python, followed by visualization in a Plotly Dash (https://dash.plotly.com/) web application. Finally, the processed outputs were compiled and formatted into a PDF report to provide stakeholders with a concise, high-level overview. Figure 3 visualizes this process.

During the RADIAL trial, a structured set of KPIs specific to the helpdesk was systematically monitored to evaluate issue tracking and KB usage. These KPIs were incorporated into both the monthly and cumulative reports (on-demand), with all metrics contextualized relative to their reporting periods. Table 1 summarizes the collected KPIs.

To analyze KB usage patterns, the same data extraction and processing workflow used for ticketing metrics was applied. Post-study, a KB usage survey summarized key measures including KB search activity across the full reporting period, total searches, mean searches per day, and account-level breakdowns by country, CRA, and support team. In the KB, the fundamental unit of content was the article; therefore, the survey also reported total and mean daily article views, along with rankings of the most frequently viewed articles.

To analyze KB usage patterns, the same data extraction and processing workflow used for ticketing metrics was applied. Post-study, a KB usage survey summarized key measures including KB search activity across the full reporting period, total searches, mean searches per day, and account-level breakdowns by country, CRA, and support team. In the KB, the fundamental unit of content was the article; therefore, the survey also reported total and mean daily article views, along with rankings of the most frequently viewed articles.

**Table 1:** Key Performance Indicator (KPI) Collection

Category	Data Collected	Calculation / Reporting Rules
Ticket Table Fields	Ticket ID, Site ID, Status, Priority, Assigned Type, Creator Contact Info, Assigned Agent, Subject, Creation Date, Total Replies, Date of Last Reply, Ticket Age	N/A
Monthly Counts	Number of tickets with activity in the month	Includes all tickets active in reporting month, regardless of creation date or status
Ticket Age	Mean age, age distribution	Resolved/Closed: days between creation and resolution; Unresolved: days between creation and reporting month end
Ticket Distribution	By Type, Site, Status, Priority Level	Count of tickets in each category
Reply Metrics	Total replies per ticket, Average replies per ticket	Calculated per reporting month
Agent Workload	Tickets assigned per agent	Count of active or resolved tickets per agent
KB Usage	Frequency of free-text search terms	Top 20 terms each month; Top 15 across full reporting period

KPIs were collected for the duration of RADIAL

## Results - Key Metrics from the RADIAL Trial

The subsequent sections present descriptive data and observations drawn from the support ticketing system and KB utilization patterns, supplemented by insights from the global issue tracker. These datasets served as monitoring instruments of the RADIAL technology support system effectiveness, supporting a more comprehensive understanding of system usage and operational trends. All metrics are reported for the full period from 1 July 2023-1 December 2024, during which the first site was activated on 10 July 2023, the first participant was enrolled on 23 August 2023, and the final participant was enrolled on 10 June 2024.

### Knowledge Base Usage Analytics

**Fig. 4:**
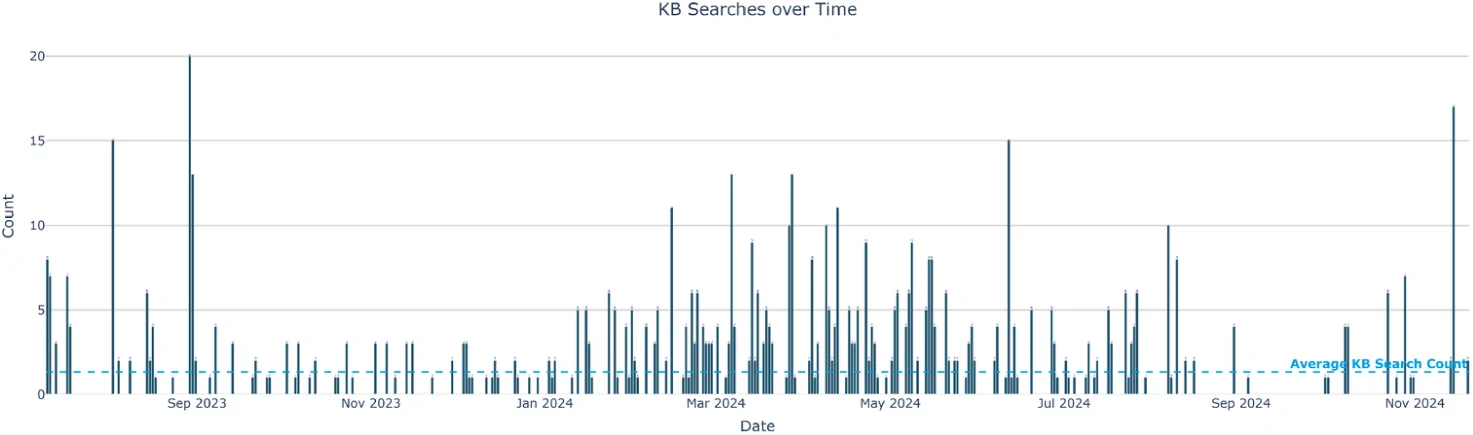
Distribution of 696 RADIAL KB searches over the 17-month reporting period; 1.3 average KB searches per day

A total of 696 searches were conducted through the RADIAL KB free-text field, averaging 1.3 searches per day over the reporting period. As shown in Fig. 4, search activity peaked between February and September 2024, mirroring the ticket submission pattern (Fig. 6), with additional increases in September 2023 and November 2024. Searches from seven distinct KB user accounts were analyzed; UK sites accounted for the highest proportion (n = 261; 37.5%), while there were no searches by Italian sites. The KB free-text search was also frequently consulted by the support team and Spanish sites, whereas Danish and German sites showed lower search activity. When normalized by number of sites or number of participants, UK sites again demonstrated the highest search activity (Table 2).

**Table 2:** Knowledge Base (KB) Search Activity

User Account	Total Search Count	Number of Sites	Searches per Site $^{1}$	Number of Participants	Searches per Participant $^{2}$
United Kingdom	261	9	29	22	11.9
Spain	98	10	9.8	27	3.6
Poland	41	8	5.1	25	1.6
Denmark	11	3	3.7	5	2.2
Germany	6	3	2	15	0.4
Italy	0	5	0	14	0
Support Team	209	N/A	N/A	N/A	N/A
CRAs	44	N/A	N/A	N/A	N/A

Distribution of KB searches by user account, site-normalized$^{1}$ and participant-normalized$^{2}$ search activity; CRAs, Clinical Research Associates $^{1}$ 8.3 average KB searches per clinical site through the KB free-text field $^{2}$ 3.3 average searches per participant through the KB free-text field

The three most frequently searched terms were all related to the smart injector cap (n = 58; 8.3%), followed by the glucometer (n = 46; 6.6%) and a third-party vendor app associated with the smart injector cap (n = 22; 3.1%) that transferred insulin dosing data from the study app to the study platform. Other terms with more than ten searches included those related to study visit 6 (telehealth visit for remote study arm), Randomization and Trial Supply Management System (RTSM), and the RADIAL study app.

**Fig. 5:**
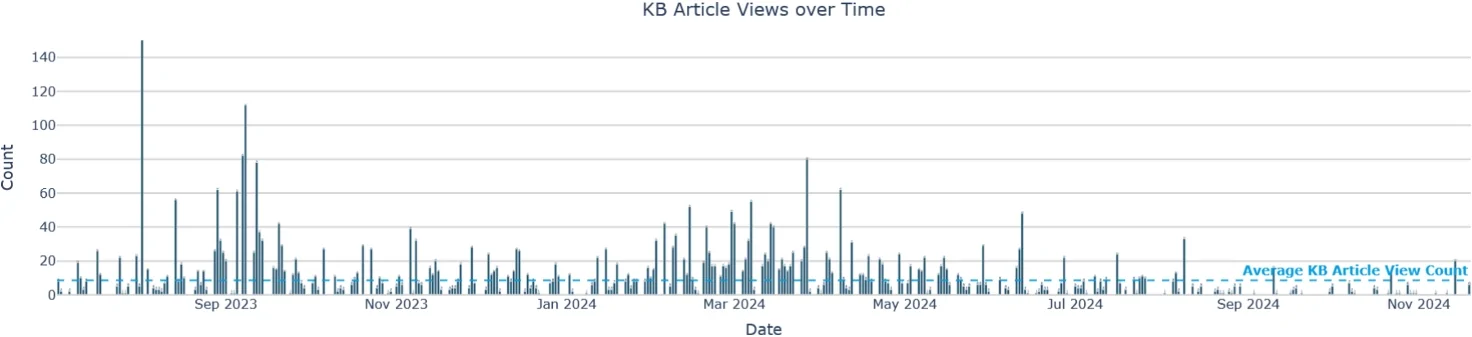
Distribution of 4350 article views over the 17-month reporting period; 8.6 average article views per day and 51.2 average views per article

A total of 4350 article views were recorded across 85 active articles in the RADIAL KB during the reporting period, averaging 8.6 views per day and 51.2 views per article. Articles were organized into five main folders – FAQs, Vendors, Site/CRA Training, Participant Training, and Other Categories – each containing multiple subcategories, with individual articles often linked to more than one category. As shown in Fig. 5, view counts peaked in September 2023 and again from February to June 2024, followed by a decline toward the end of the reporting period. In the UVdesk open-source version, KB user accounts were not associated with article view, preventing user group-specific analytics.

The most frequently viewed articles included the training materials provided to participants and sites, namely “Participant Training Booklet 1” for participant training (n = 284; 6.5%), “Site training: The RADIAL Study Portal and Application” (n = 255; 5.9%), “Site training: IMP and Study Material Management & Logistics” (n = 220; 5.1%), “Site training: RADIAL eCRF User Manual” (n = 194; 4.5%) for site training, and “Participant Training Booklet 2” for participant training (n = 185; 4.3%). Additional articles exceeding 100 views featured troubleshooting guides, FAQs, and videos for the smart injector cap and associated app, instructions for pairing and using the glucometer, the study team Electronic Data Capture (EDC) user guide, visit checklists for the conventional arm, and the RADIAL RTSM site user transaction manual.

### Ticket Monitoring and Evaluation

**Fig. 6:**
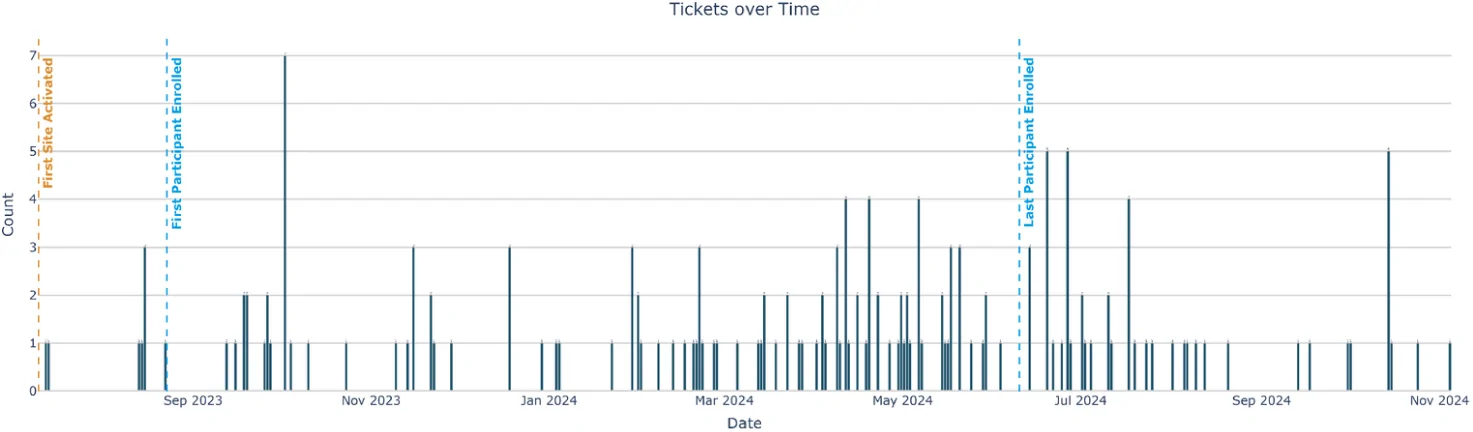
Distribution of 169 ticket submissions over the reporting period; 9.9 average tickets per month

During the RADIAL trial, a total of 169 tickets were submitted to the RADIAL helpdesk across 29 of the 38 participating sites. Ten sites did not enroll participants, but three of them still submitted tickets, indicating that approximately 90% (n = 26) of sites with participants engaged with the support system at least once. An internal identifier was designated for tickets raised by clinical operations or the support team not attributed to a single site. This category accounted for 16% (n = 27) of all submissions. As shown in Fig. 6, ticket submissions peaked between February 2024 and September 2024, coinciding with the period of highest number of participants actively enrolled in the trial. Ticket distribution per country, along with corresponding participant numbers and ticket-to-participant ratios, is summarized in Table 3.

**Table 3:** RADIAL helpdesk ticket counts

Group	Total Ticket Count	Number of Participants	Tickets per Participant$^{1}$
Clinical Operations / Support Team	27	N/A	N/A
Denmark	6	5	1.2
Germany	13	15	0.9
Italy	16	14	1.1
Poland	41	25	1.6
Spain	23	23	1
United Kingdom	43	21	2.1

Ticket counts per country relative to participant numbers; Three sites without enrolled participants also submitted tickets. Sites that enrolled participants but did not submit tickets are not represented.$^{1}$Sites with participants enrolled submitted an average of 1.3 tickets per participant

Resolution times varied between tickets, with 39 tickets (23.1%) resolved within 24 hours and approximately half (n = 85; 50.3%) resolved within three days. Across all tickets resolution ranged from a few hours to 140 days, with a mean ticket resolution time of 14.6 days.

The most common ticket type concerned device-related issues (n = 77; 45.6%), including problems with the glucometer, smart cap for insulin dosing, and their Bluetooth pairing with smartphones. This was followed by study app-related issues (n = 29; 17.2%) and requests for live support or standby (n = 15; 8.9%). Remaining tickets (n = 48; 28.4%) addressed general support, study platform issues, telehealth, RTSM and logistics, account creation, and onboarding. Notably, the ticket type “Schedule Live Support Session” was introduced in February 2024.

At study completion, 157 tickets were classified as “Resolved” and 12 as “Closed”, indicating that 7.1% of submitted tickets were unrelated to the RADIAL trial according to the status convention. Ticket priority levels remained largely at the default “Low” (n = 166; 98.2%), with only one ticket classified as “Medium” and two as “High”.

Most tickets (n = 115; 68%) were resolved with 2 to 6 replies, and 21.3% (n = 36) with three replies; the number of replies ranged from 1 to 30, with a mean of 5.6 per ticket.

Slightly more than half of all tickets (n = 86; 50.9%) were resolved by a single ticket agent who served on the RADIAL support team for the entire study, and 75.7% (n = 128) were resolved by three agents who remained throughout the trial. In total, 13 agents resolved or closed at least one ticket.

Detailed post-processing of the global issue tracker revealed several qualitative trends. Most tickets were recategorized as “Technical Support”, followed by “Process-Related”. The overall ticket count may be reduced by 10%–20% when consolidating multiple submissions related to the same issue. Device-related tickets were further reviewed to distinguish between smart injector cap and glucometer issues, noting that some categories (e.g., “Internal Reporting”, “Glucometer”) were introduced midway through the reporting period. Approximately 20% of device-related issues involved both devices, while 45% concerned only the glucometer and 35% only the smart injector cap. However, it should be noted that the glucometer was used by all participants and the smart injector cap only by participants in the hybrid and remote arm. In early June 2024, issues involving either device were escalated to third-party vendors due to the risk of losing critical participant-reported study data.

The distribution and frequency of KB searches (Fig. 4) and KB article views (Fig. 5) were higher during the recruitment phase and decreased on average toward the end of the trial. A similar pattern was observed for ticket submissions (Fig. 6). Individual peaks frequently coincided with (upcoming) study visits or technical issues that could potentially lead to the loss of participant-reported outcome data.

## Recommendations for a Technology Support Framework in DCTs

The subsequent sections provide general recommendations on functional components, configuration and operational considerations for building an effective technology support system in DCTs based on the experiences with RADIAL and in line with what was found in literature [7, 15, 17, 18].

### Core Functional Components of a DCT Technology Support System

A DCT technology support or helpdesk system comprises three primary components: a ticketing system in the backend, a KB in the frontend, and a technology-support meeting module. The technology support system can be implemented as a stand-alone system or integrated into another study platform, as will be discussed in Section “Discussion”.

The usage analysis (Section “Knowledge Base Usage Analytics”) revealed frequent engagement with the KB, suggesting that the frontend was well used and appeared to function as a central information hub that supports trial operations. This encompasses all training and educational materials on the study and technologies/systems used, as well as the incorporation of FAQs. In RADIAL, the frontend was designated as the KB. Users should have the opportunity to both browse and search for specific information. The implementation of a chatbot could offer a viable additional feature as further discussed in Section “Discussion”. Finally, in cases where users are unable to locate the desired information, it is imperative to provide an avenue for them to submit a request (i.e., create a ticket) and establish direct communication with a human (i.e., designated ticket agent). In order to ensure precise and complete issue descriptions, it is advised to employ structured and issue type-specific submission templates.

The backend should feature a dashboard for ticket agents to manage and respond to support requests efficiently. Furthermore, the backend should be equipped with a Content Management System (CMS) for the KB that allows content to be extended and updated. Additionally, the backend should contain an analytics and reporting module that facilitates the extraction of quality and performance control metrics. In an ideal scenario, certain workflows (e.g., email notifications) can be configured as automated backend processes. The management of user access, permission, and roles should be conducted through the system. The implementation of a form builder capable of generating structured forms tailored to specific ticket types could also prove advantageous.

**Fig. 7:**
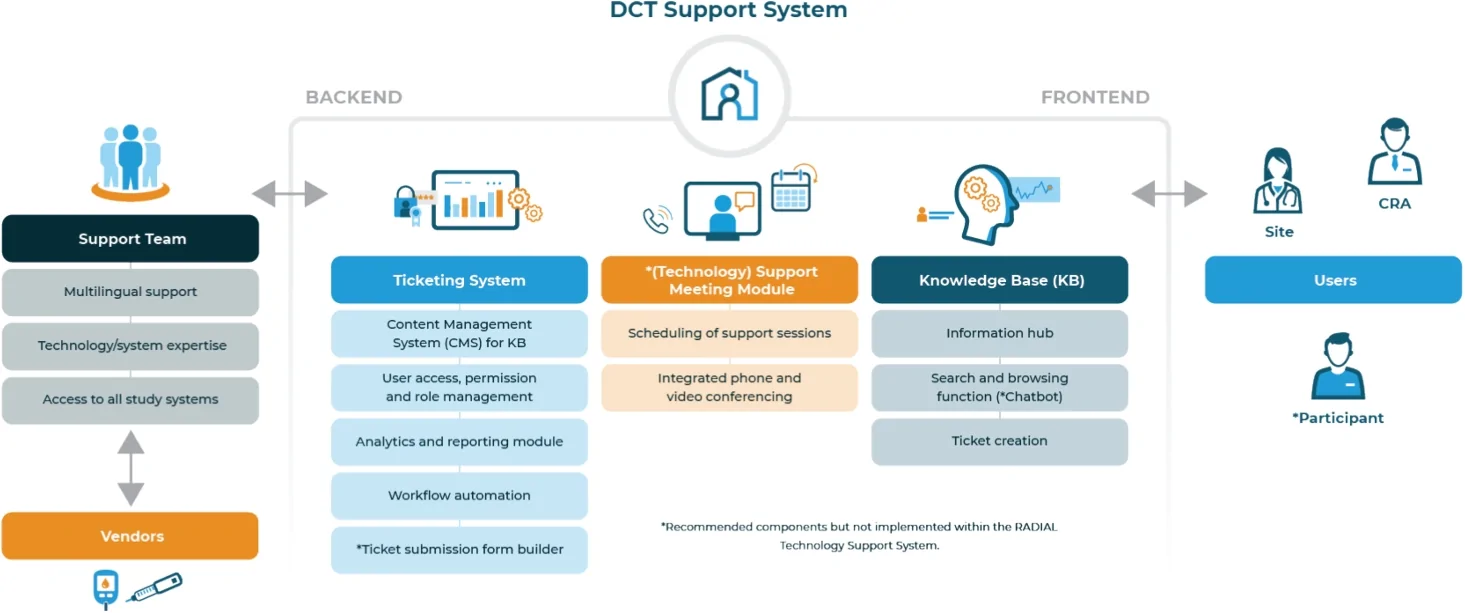
Implemented technology support system components along with recommended additional modules based on the experiences with RADIAL (https://trialsathome.com/technical-support-system/)

Approximately six months into the support phase, the demand for both immediate live assistance and structured standby support became increasingly apparent, leading to the introduction of a dedicated ticket type “Schedule Live Support Session”. Within the RADIAL framework, real-time support was primarily delivered via telephone or video calls, requiring third-party providers. To avoid reliance on external providers, and ensure greater data privacy, the integration of a technology support meeting module is recommended as a third component. Such a module should enable users to schedule remote support sessions directly with a support team member while offering functionality to specify preferred language and available time slots for support meetings. Figure 7 outlines all recommended components of an effective technical support system in DCTs based on the experiences with RADIAL.

### Set-up and Key Operational Considerations

The development of an effective technology support system is contingent upon close collaboration between relevant stakeholders. Early engagement of the support team, sponsors, and study team is instrumental in laying the foundation for a robust technology support system that can adapt to the evolving needs of a DCT study seamlessly. One of the primary questions that should be clarified early concerns the configuration of support system user journeys (communication chain) and target user group. This process entails mapping scenarios and developing template workflows for each. For system design and integration it is critical to determine whether the support system is accessible directly to study participants or whether issues are first routed through site staff supported by CRAs before reaching the helpdesk. In RADIAL, tickets were initiated by clinical site personnel or CRAs either in response to an individual participant’s question or issue, or to address overarching site issues (affecting multiple participants) that could not be resolved through browsing the KB. Ticket creation was always independent of the trial arm. The KB was accessible to all users at any time; therefore, no specific initialization event for KB searches was required.

In RADIAL, the open-source UVdesk solution was used and configured to meet the project’s specific requirements; therefore, no software licensing costs were incurred. One lead developer was responsible for setting up and deploying the system and database, as well as customizing UVdesk to the needs of RADIAL. Within the RADIAL research project, the technical support system was hosted by a consortium member; however, in a typical setting, an external hosting provider would be required, resulting in ongoing server maintenance costs. The developer remained available on demand throughout the entire trial, and dedicated time was allocated for KB content management and user acceptance testing prior to operating the system in a production environment.

The RADIAL Helpdesk operated from 9:00 a.m. to 6:00 p.m. on weekdays, without 24/7 support. On average, seven agents were actively involved in helpdesk operations, each covering different time slots depending on resource availability. Additional personnel resources were required for governance and reporting activities. Although this setup represented the lower end of the budget spectrum, it was sufficient for the RADIAL proof-of-concept study; however, for larger-scale implementations, resource requirements for (technical) support should not be underestimated.

In RADIAL, ticket distribution among agents reflected substantial heterogeneity in experience and continuity within the support team. A proposed measure to build a highly skilled support team is to involve them from the outset of the DCT design, development, configuration and testing phase. Proactive engagement ensures the identification of potential technical challenges well before deployment, reducing inefficiencies and improving system usability and user satisfaction. A dedicated support team should possess broad expertise in study processes and technology-related knowledge, enabling them to competently resolve or redirect support requests. Support agents should also have access to, and be proficient users of, all relevant systems used within the trial. Furthermore, it is advised to have at least one native speaker per language used within the trial to guarantee support and accessibility in multiple languages.

Ticket type distribution in the RADIAL trial underlines that from a clinical trial operations perspective, sites and CRAs cannot always be fully familiar with the intricacies of the multiple technical integrations involved. Given the extensive interconnectivity across platforms and vendors, identifying the root cause of operational or technical issues is often challenging even for the support team. As such, effective oversight necessitates the involvement of dedicated team with a comprehensive view and understanding of the technological landscape, ensuring coordination and continuity across all systems and stakeholders.

It is imperative to highlight the pivotal role of well-defined internal (within the support team) and external (as a conduit to third-party vendors, CRAs) workflows as well as clear communication channels for both routine and urgent inquiries. These workflows are essential to enhance interdisciplinary communication and collaboration, and ensure a scalable, reliable, and responsive support framework across all stakeholders.

The continuous improvement of support efficiency and user engagement can, for example, be achieved by establishing active follow-up procedures to clarify incomplete or ambiguous tickets through direct queries or phone calls, ensuring timely issue resolution. Site staff and CRAs should be encouraged to utilize the KB prior to submitting tickets. This can be achieved by incorporating self-troubleshooting guidance into onboarding materials and refresher training sessions. Ticket quality should be subject to regular monitoring. On the one hand, feedback to users on how to improve issue reporting can be given to reduce resolution delays and avoid unnecessary escalations. On the other hand, feedback from users should be collected, for example, on whether the KB contained the needed information. Such feedback supports the continuous updating and refinement of published content.

Optimization of ticketing system governance requires a daily ticket review to identify critical to quality issues and ensure proper tracking.

To ensure timely resolution of issues related to third-party technology, proactive management and logging of such issues into vendor ticketing systems is recommended. Furthermore, it is advised to track vendor ticket processing times and incorporate vendor response time data in governance meetings to escalate high-impact issues and improve vendor accountability.

A structured process for tracking helpdesk performance metrics should be implemented. Insight derived from ticket data can be used to identify gaps in usability, support processes, and training. Performance metrics should also inform refinements to training materials and updates to the KB based on frequently reported issues and common troubleshooting patterns. Periodic reviews of helpdesk efficiency should be conducted to optimize workflows and ensure alignment with study requirements.

## Discussion

A central technical consideration concerns the optimal integration of a support system within the clinical trial framework. In practice, three main approaches can be distinguished: 1) Embedding within a single system component (e.g., EDC or site portal). This option streamlines access for that component and facilitates limited data consolidation. However, in the context of DCTs, it risks being too narrow, since many issues arise outside of the EDC (e.g., devices, telehealth, logistics, mobile apps). Without broader integration, users may still need multiple access points and the helpdesk itself becomes fragmented and inefficient in maintaining oversight. 2) Embedding across an integrated trial platform using single sign-on (SSO) and an identity and access management (IAM) such as Keycloak (https://www.keycloak.org/). When the support system is integrated at platform level, spanning multiple components (EDC, RTSM, apps, telehealth, logistics), SSO can provide unified access and efficient role management. This eliminates the problem of multiple logins while still delivering the interoperability benefits of integration. The trade-off is that it requires more development resources and coordination between vendors. 3) Deploying a standalone helpdesk. This option offers independence from individual systems, continuity during platform outages, and broader coverage across trial interfaces. It is flexible but may introduce additional user management overhead if not linked to an IAM solution. The choice among these strategies is shaped by development resources, user requirements, and the balance between implementation complexity and integration benefits [4]. For smaller trials such as RADIAL, where the number of collaborators is manageable, general country-level login credentials to access the KB – serving as a central hub linking to other systems – and additional granularity through internal role allocation for the backend were sufficient. However, for larger and more complex studies, embedding within an IAM-enabled platform or deploying a standalone helpdesk with SSO integration may represent more sustainable solutions [3].

To support the RADIAL trial, a standalone helpdesk was implemented, employing separate country-level login credentials for the frontend and individual credentials for the backend. The helpdesk acted as a gatekeeper to prevent clinical sites from independently determining which third-party vendor helpdesk to contact for specific issues. Sites were instructed to engage with third-party helpdesks only under exceptional circumstances and solely when advised by the central RADIAL helpdesk. Furthermore, given the integrated architecture of the RADIAL system, the root cause of technical issues was often multifactorial, and single vendors generally did not possess the capacity to independently resolve cross-system or overarching issues.

From a user’s perspective, one interface-related consideration became apparent: The ticket request form lacked an option to directly add additional e-mail addresses of collaborators. In RADIAL, this limitation was addressed by pre-filling the message text with the instructions to manually include additional e-mail addresses at the beginning of the message. Ticket agents appended the respective addresses to the ticket record. Although functional, this approach was error-prone and adopted mainly to avoid changing the user interface mid-study. For future trials, a dedicated input field for additional recipients in the ticket request form would present a more robust solution.

An additional consideration concerns the uneven patterns of KB engagement observed across countries. For example, UK sites recorded 261 KB searches while Italian sites recorded none, with other countries falling in between. While such variation could be linked to site size, staffing resources, or differences in technical literacy, all sites had been assessed as technically capable before contracting. This suggests that disparities in KB usage may reflect local working practices or preferences for direct communication rather than limitations of feasibility. From an operational standpoint, this diversity in engagement is not necessarily problematic as long as site responsibilities are met. However, from a support-system perspective, it underscores the importance of monitoring usage patterns, since uneven adoption can influence workload distribution across CRAs and support staff, and may affect the consistency of oversight.

The assumed preference for direct communication with the helpdesk among Italian sites was not supported by an increased number of ticket submissions. Instead, the comparatively low engagement levels may be attributed to a language barrier with German and Danish sites showing similar patterns. English served as the primary communication language in RADIAL; KB articles were provided in English by default, and the option to switch to other languages was likely not sufficiently visible. Additionally, nearly all submitted tickets were written in English, indicating that the language barrier may have limited helpdesk engagement at some non-native English-speaking sites. It should also be noted that the absence of recorded KB search counts does not necessarily imply that the respective sites did not utilize the KB. Rather, it indicates that users did not engage with the free-text search functionality. This observation may also suggest that CRAs were increasingly using the KB search in these countries. However, definite causal relationships cannot be established, as the CRA account activity data and article-view metrics were not disaggregated at the country level.

Multilingual accessibility features, and multilingual support (documentation and via agents) should therefore be considered as success factors and treated as core design requirements of a DCT support especially in multi-country trials. KB materials, and live support available in all trial languages directly affect inclusivity and equity of participation. Accessibility could also extend beyond just translation and include plain-language instructions, visual aids, and alternative formats to accommodate differences in health and digital literacy based always on the trial population.

In RADIAL, more than half of all tickets were resolved by a single agent. This raises the risk of dependency on individual staff and potential single-point failures. In a relatively small proof-of-concept trial like RADIAL such a workload distribution may be tolerable, however, in larger multi-country trials greater redundancy would be required. Automated workflows – such as template replies or chatbot-assisted triaging – offer a scalable strategy to handle routine issues and to balance workload across agents. Such measures improve sustainability, reduce the risk of bottlenecks, and ensure continuity in the event of staff turnover or (unexpected) absence.

The functionality of a chatbot can range from a rule-based engine that provides guidance to users during the troubleshooting process for common issues, to a large language model integrated with a retrieval-augmented generation (RAG) approach, where study materials are indexed and dynamically retrieved at query time. This allows the system to address open-ended questions without the need for training a dedicated model. Compared with fine-tuning approaches, which may increase domain specificity but require additional validation and maintenance, RAG-based systems can more readily ensure that responses are grounded in validated and up-to-date trial documentation. Incorporation of a chatbot has the potential to reduce the workload of the support team (by avoiding repetitive work involving the same issues for ticket agents) while promoting self-mitigation strategies, provided that risks related to incomplete retrieval, ambiguous source material, or hallucination are mitigated through validation of source content, transparency of information provenance, and escalation to human agents where necessary.

While the BYOD approach offers advantages for participants – allowing engagement through their personal mobile devices – it introduced significant challenges for the support team in RADIAL. Accommodating diverse hardware and software configurations substantially increases the demand for technical support resources, with troubleshooting compatibility issues scaling in proportion to device heterogeneity. Rather than framing this as a burden, BYOD can instead be viewed as a design principle that necessitates flexible, device-agnostic support systems [9]. By anticipating variability in technology from the outset, such systems enhance scalability and resilience, particularly as DCT methodologies are adopted more widely. Complementing this, governance processes must ensure that device diversity (perhaps not only smartphone), including software updates, do not compromise validation, data privacy, or cross-site standardization.

Ensuring data privacy was a central priority in RADIAL. To mitigate risks, a constant message was broadcasted via the KB, ticket creation forms, and ticket submission confirmation emails reminding users not to include personal data such as addresses or phone numbers. In the rare event of a breach, immediate escalation procedures were activated, requiring the removal of compromised data from databases, email systems, servers, and backups. This incident occurred on a single occasion, when a participant’s private address and phone number were inadvertently disclosed.

Following the trial, qualitative interviews were conducted with site staff. The detailed results will be reported in a separate publication. However, it is relevant to mention that some site representatives expressed a preference for direct helpdesk-participant interaction to streamline information flow and reduce procedural complexity. Nonetheless, routing inquiries through site personnel and CRAs can serve as a protective buffer for participant safety. Direct communication channels may be suitable for procedural matters such as system access, whereas critical cases involving participant health should remain mediated. Thus, the optimal communication pathway depends on both the issue type and the broader study context.

Due to the unequal distribution of participants across trial arms – particularly the small cohort in the fully remote arm (n = 8) – KPIs, including arm-specific measures, were not analyzed separately for the conventional, hybrid, and fully remote approaches. Furthermore, capturing trial-arm identifiers within the KB and ticketing system logs would have required additional technical customization of the open-source UVdesk solution, which was not included in the initial setup but should be considered in future implementations. Stratified analyses could yield operational approach-specific insights and inform the optimization of future trial design and conduct.

In RADIAL, KPI reports were generated monthly as PDFs following SQL-based data queries. Alternatively, web applications such as Plotly Dash can be configured for direct database access and deployed on servers, allowing sponsors and stakeholders to monitor predefined support system KPIs in real time. Both approaches provide flexibility for extending the KPI set as needed. Beyond descriptive reporting, DCT support systems could also integrate a forward-looking dimension by using KPIs as early warning indicators. For example, a sudden spike in resolution times might signal systemic issues such as vendor performance, a training gap or site understaffing. Leveraging predictive analytics on historical ticketing data (e.g., ticket spikes pattern like before or after site activations) could help forecast peak support demands, anticipate recurring technical issues, and proactively allocate resources. This transition from descriptive to predictive monitoring would strengthen both operational resilience and trial oversight.

## Conclusion

Successful technical support in DCTs requires seamless workflow integration, intuitive design, and alignment of clinical needs and technical requirements, sustained by continuous feedback, adequate resource allocation, and adaptable communication structures. Holistic insights from RADIAL may inform the planning, design, implementation, and operation of future technology support systems in DCTs, guiding trialists and support teams toward more effective practices.

## Data Availability

All data supporting the findings of this study are available within the paper.
